# Supplementary material for: Glucocorticoid Receptor Isoforms in Breast Cancer Raise Implications for Personalised Supportive Therapies
Source: Int J Mol Sci. 2024 Nov 3;25(21):11813. doi: 10.3390/ijms252111813 (PMC11546579; doi:10.3390/ijms252111813)
Supplement: Supplementary file 1 [file ijms-25-11813-s001.zip › ijms-3232238-supplementary.pdf]

Supplementary Table S1. Samples histological classification

**Histological types**	**n**
Invasive ductal carcinoma	165
Invasive lobular carcinoma	15
Mucinous carcinoma	2
Metaplastic carcinoma	1
Medullary carcinoma	1
Apocrine carcinoma	3
Mixed (ductal with mucinous/neuroendocrine/micropoapillary components, lobular with ductal/apocrine components)	7
total	194

Supplementary Table S2. Antibodies and their dilution used for routine pathological characterisation

Antibody	Product name	Manufacturer	Catalogue number	Clone	Dilution
ER	Confirm anti-ER (SP1)	ROCHE	5278406001	SP1	RTU
PR	Confirm anti-PR (1E2)	ROCHE	5277990001	1E2	RTU
Her2	Pathway anti-HER-2/Neu (4B5) Rabbit mono	ROCHE	5278368001	4B5	RTU
Ki67	Ki-67	Agilent (Dako)	M724001-2	MIB-1	1:100
DDISH	VENTANA Her2 Dish DNA PRB CKT-US Export	ROCHE	8314373001		RTU

RTU: ready-to-use, antibodies are "prediluted," meaning the vendor has previously established the optimal dilution of the antibody; ER: oestrogen receptor; PR: progesterone receptor; Her2: Receptor tyrosine-protein kinase erbB-2; Ki67: Antigen Kiel 67; DDISH: Dual-color dual-hapten in situ hybridization
